# CD205 (DEC-205): A recognition receptor for apoptotic and necrotic self

**DOI:** 10.1016/j.molimm.2008.11.016

**Published:** 2009-03

**Authors:** Rachel E. Shrimpton, Matt Butler, Anne-Sophie Morel, Efrem Eren, Swee Shan Hue, Mary A. Ritter

**Affiliations:** Department of Immunology, Division of Medicine, Imperial College London, Hammersmith Campus, London W12 0NN, United Kingdom

**Keywords:** Dendritic cells, Apoptosis, CD205, DEC-205

## Abstract

CD205 is an endocytic receptor that is expressed at high levels by cortical thymic epithelial cells and by dendritic cell (DC) subsets, including the splenic CD8+ DC population that is responsible for cross-presentation of apoptotic cell-derived antigens. Antigen endocytosed via CD205 enters the MHC class I and MHC class II antigen presentation pathways and is subsequently presented to both CD4+ and CD8+ T cells. Despite the known role of CD205 in antigen uptake, the nature of the ligands bound by CD205 has not been determined, and most studies have relied on the use of monoclonal antibodies as surrogate ligands. To go beyond this approach, we created a panel of CD205–IgG fusion proteins spanning the extracellular portion of CD205 and used these to identify the physiological distribution of CD205 ligands. Our data demonstrate that two areas of the CD205 molecule, within C-type lectin-like domains (CTLDs) 3 + 4 and 9 + 10, recognise ligands expressed during apoptosis and necrosis of multiple cell types, and are additionally expressed by live cells of the dendritic cell line DC2.4. Thus, CD205 acts as a recognition receptor for dying cells, potentially providing an important pathway for the uptake of self-antigen in intrathymic and peripheral tolerance.

## 1 Introduction

CD205 is an endocytic type I C-type lectin-like molecule consisting of a single polypeptide chain; the extracellular portion contains an N-terminal cysteine-rich domain (CyR)^1^, a fibronectin type II domain (FnII), and 10 domains structurally (although not necessarily functionally) resembling C-type lectin domains (C-type lectin-like domains, CTLDs) (East and Isacke, 2002; Jiang et al., 1995; McKay et al., 1998). This structure places CD205 in the Mannose Receptor (MR) family (East and Isacke, 2002). The MR itself has been well characterised, and contains two distinct ligand binding sites; CTLD4 is a true C-type lectin that binds terminal mannose, fucose, *N*-acetylglucosamine and glucose (Taylor et al., 1992), whereas the CyR is a lectin that binds terminal sulphated GalNAc-4-SO4 (Fiete et al., 1998). Although structurally related, CD205 does not contain the conserved residues required for lectin and C-type lectin binding (East and Isacke, 2002), and the ligands bound by CD205 have remained elusive.

The roles of CD205 in antigen uptake, processing and presentation have however been well characterised. CD205 is expressed at high levels on cortical thymic epithelium, thymic medullary DCs (CD11c+ CD8+), and subsets of peripheral DCs (CD11c+ CD8+ splenic/lymph node DCs, dermal/interstitial DCs, and Langerhans cells) (Heath et al., 2004; Jiang et al., 1995). Studies of CD205 function have mostly relied on the use of monoclonal antibodies as surrogate ligands, and have demonstrated that when antigen is targeted to DCs by conjugation to an anti-CD205 antibody, the antigen is endocytosed, processed, and presented on both MHC class II and MHC class I molecules (cross-presentation) with high efficiency (Bonifaz et al., 2002; Mahnke et al., 2000). The recycling properties of CD205 are responsible for this effect, which are in turn guided by motifs within the intracellular tail of CD205. A tyrosine containing FSSVRY motif is responsible for initial clathrin-coated pit-mediated endocytosis, while an acidic EDE triad motif targets CD205 to late endosomes/early lysosomes, allowing endocytosed antigen to reach the MHC class II loading machinery (Mahnke et al., 2000).

The ability of CD205 to deliver antigen to DCs for presentation on MHC class II and cross-presentation on class I has generated much interest in the use of anti-CD205-antigen conjugates as therapeutic reagents. When antigen is delivered to DCs *in vivo* via CD205 without an inflammatory stimulus, tolerance to the antigen is induced (Bonifaz et al., 2002; Hawiger et al., 2001). This occurs by inducing deletion and unresponsiveness (anergy) in antigen specific CD4+ and CD8+ T cell populations, and the induction of regulatory T cell subsets (Mahnke et al., 2003). CD205 is therefore an attractive target for tolerisation to autoantigens, and has been used to this effect to prevent the onset of diabetes in a mouse model (Bruder et al., 2005). Conversely when a maturational stimulus is co-administered with CD205-targeted antigen, long-lived immunity via antigen-specific CD4 and CD8 T cells results (Bonifaz et al., 2002, 2004; Hawiger et al., 2001). This has resulted in successful vaccination against HIV gag-antigens and cancer antigens in murine disease models (Bozzacco et al., 2007; Mahnke et al., 2005; Trumpfheller et al., 2006).

It has thus become clear that CD205 plays an important role in antigen uptake for presentation and cross-presentation to T cells; indeed, because antigen uptake via CD205 in the steady-state results in tolerance, this suggests that CD205 plays an important role in CD4 and CD8 T cell tolerance induction to self-antigen both in the periphery and in the thymus (Jiang et al., 1995). Given that CD205 can deliver antigens to the cross-presentation pathway, and that CD11c+ CD8+ CD205+ DCs are specialised for the cross-presentation of apoptotic cell-derived antigens (Heath et al., 2004; Iyoda et al., 2002; Liu et al., 2002; Steinman et al., 2000), we hypothesised that CD205 may act as a recognition receptor for the uptake of ‘self’ in the form of apoptotic cells. To test this hypothesis, we constructed a panel of CD205–IgG fusion proteins spanning the extracellular domains of the molecule. These fusion proteins were used to test whether CD205 could bind apoptotic cells, and to identify the regions of the molecule responsible for such ligand binding. Our data demonstrate that CD205 does indeed recognise cells that are undergoing apoptosis and necrosis, and that CD205 ligands are additionally expressed by live cells of the cloned DC cell line DC2.4. Thus, CD205 may provide a mechanism for uptake and presentation of self-antigens for intrathymic and peripheral tolerance induction.

## 2 Materials and methods

### 2.1 Animals

Male and female C57BL/6 and BALB/c mice were purchased from Harlan and maintained in the Biological Services Unit at the Hammersmith Campus of Imperial College London. Mice were sacrificed at 2–6 months of age and the thymus and hind limb bones removed. All animal work was performed in accordance with UK Home Office regulations.

### 2.2 Cell lines and culture media

A20 B cells, Chinese hamster ovary (CHO) cells, JAWS II (all from the American Type Culture Collection) DC2.4 (a kind gift from Kenneth L Rock) and the F1 cortical thymic epithelial cell line (Spanopoulou et al., 1989) were cultured in Complete Medium (CM), consisting of DMEM (Invitrogen Life Technologies) supplemented with 10% heat inactivated FCS (Labtech International), 2 mM l-glutamine, 1 mM sodium pyruvate, 100 U/mL penicillin, and 100 μg/mL streptomycin (Invitrogen Life Technologies) at 37 °C in 5% CO_2_. Transfected CHO cells were also grown in the serum-free medium UltraCHO (Cambrex), supplemented with penicillin and streptomycin. The NLDC-145-secreting hybridoma (ATCC) was grown in serum-free AIM-V medium (Invitrogen Life Technologies). Antibody was purified from the culture supernatant using standard protein-G affinity purification techniques. The conditionally immortalised cortical thymic epithelial cell line YO1 (Williams et al., 1996) was grown at 33 °C in complete medium supplemented with 100IU/mL recombinant mouse IFNγ (Biosource). Three days prior to experiments, the cells were seeded as required and moved to 37 °C. Murine bone marrow derived dendritic cells were generated using a variation of the technique of Inaba et al. (1992). Briefly, the marrow was flushed from the hind limbs, and plated at 1 × 10^6^ cells/mL in complete medium supplemented with 20 ng/mL GM-CSF (Biosource) in culture dishes. At day 3, the adherent cells were washed three times with warm PBS and fresh medium with GM-CSF added. On day 7, the non-adherent DCs were harvested and used in uptake experiments.

### 2.3 Generation of CD205–IgG fusion protein plasmids

RNA was extracted from a murine thymus and the F1 cell line (Spanopoulou et al., 1989) using RNAsol B (Biogenesis), according to the manufacturer's instructions. cDNA was synthesised using the First-strand cDNA Synthesis Kit (GE Healthcare) according to the manufacturer's instructions. cDNA encoding paired domains of CD205 was amplified using the primers listed in Table 1 by PCR, using *Pwo* polymerase (Roche). The PCR products were ligated into the multiple cloning site (MCS) of the signal PIG plus vector (R&D Systems) after digesting both the product and vector with the relevant restriction enzymes listed in Table 1. The plasmids were sequenced at the MRC Core DNA Facility (Hammersmith Campus, Imperial College London) using the vector sequencing primers specified by the manufacturer (Forward: 5′-ATGTGTGAGGTTTGTCACAAG-3′ and Reverse: 5′-ACTCACTATAGGGAGACCCAA-3′).

### 2.4 CD205–IgG production and purification

Preparations of plasmid DNA were made using the Endofree Maxi-prep Kit (QIAgen). CHO cells were transfected using calcium phosphate. The medium was harvested after seven days, giving a source of CD205–IgG in CM. CHO cells were subsequently passaged in UltraCHO medium containing 250 μg/mL of the selective antibiotic G418 (Invitrogen). Stable cell lines secreting high amounts of CTLD1+2-IgG, CTLD3+4-IgG and CTLD9+10-IgG were subcloned by limiting dilution. CD205–IgG was purified from UltraCHO medium by affinity purification with protein-A sepharose (Sigma–Aldrich), and stored in PBS using PD10 desalting columns (GE Healthcare).

### 2.5 Measuring CD205–IgG concentration

The concentration of CD205–IgG in culture supernatants was determined by enzyme-linked immunosorbant assay (ELISA). 96-well Maxisorp plates (Nunc) were coated with mouse monoclonal anti-human IgG Fc (clone GG7 ascites fluid; Sigma–Aldrich) diluted 1:7000 in PBS, blocked with 1% bovine serum albumin (Sigma–Aldrich) in PBS, and four-fold serial dilutions of supernatants and a human IgG standard (purified from human serum; Sigma–Aldrich, UK) from 1 μg/mL were incubated on the plates for 16 h at 4 °C. The plates were incubated with biotinylated goat anti-human IgG (Vector Laboratories) diluted to 25 ng/mL in PBS, and then with 1 μg/mL streptavidin–horseradish peroxidase (HRP; Biosource) in PBS. Between each of the steps detailed above, the plates were washed three times with 0.1% Tween-20 in PBS. Plates were developed with the colorimetric substrate tetramethylbenzidine (TMB; Zymed). The reaction was stopped by adding an equal volume of 0.5 M H_2_SO_4_, and the OD_450nm_ was determined using a Titertek Multiscan Plus plate reader (Labsystems). The concentration of human IgG in the supernatants was calculated within the log-linear phase of the curve. The concentration of purified CD205–IgG was measured using a MicroBCA Kit (Pierce), according to the manufacturer's instructions.

### 2.6 Immunoblotting

For Western blot analysis, 6 ng CD205–IgG in CM was boiled for 5 min in non-reducing SDS loading buffer, resolved by SDS-PAGE using a pre-cast 4–12% gradient gel and transferred to a polyvinyl difluoride (PVDF) membrane (all from Invitrogen Life Technologies). The membrane was blocked with 2.5% non-fat milk powder in PBS, and incubated with 2 μg/mL biotinylated goat anti-human IgG (Vector Laboratories) in 0.5% non-fat milk powder in PBS. The membrane was washed three times with 0.1% Tween-20 in PBS, and incubated with extravidin-HRP (Sigma–Aldrich) diluted 1:1000 with 0.5% non-fat milk powder in PBS. The membrane was washed again, and developed using the ECL Western Blotting System (GE Healthcare) and exposure to Hyperfilm (GE Healthcare). For dot blot analysis, 100 ng purified CD205–IgGs in a total volume of 1 μL PBS was dried onto a nitrocellulose membrane (Millipore). The blot was developed as detailed above, but using 1 μg/mL NLDC-145 (or 12G8 antibody as an isotype control; produced in-house from hybridoma supernatant) and rabbit anti-rat-HRP (1:2000 dilution; Dako Cytomation).

### 2.7 Induction of apoptosis and necrosis

A single cell suspension of thymocytes was made in CM by teasing from a thymus using 23G needles, and filtering through a 70 μm mesh. Single cell suspensions of thymocytes and cell lines (detached using Versene (Cambrex) were adjusted to 5 × 10^6^/mL. To induce apoptosis, thymocytes were incubated in tissue culture flasks for 16–20 h either at 4 °C until use (no treatment), or at 37 °C and 5% CO_2_ with medium alone, or including 1 μM dexamethasone (Sigma–Aldrich), or 5 μg/mL anti-Fas antibody (clone Jo2, BD Bioscience). To induce necrosis, cell suspensions were incubated at 56 °C for 30 min.

### 2.8 Flow cytometry

For single colour flow cytometry assays, 3 × 10^5^ cells were resuspended in 200 μL of PBS containing 1–10 μg/mL purified fusion protein, 2.5 mM CaCl_2_, and 5% FCS. The cells were incubated on ice for 2 h, then fixed by the addition of 100 μL 4% w/v paraformaldehyde in PBS directly onto the staining reaction. The cells were fixed for 20 min at 4 °C, then washed three times with flow cytometry buffer (FCM buffer; ice-cold PBS containing 1% heat-inactivated FCS, 5 mM ETDA, 0.1% w/v sodium azide). Cells were then incubated with 1.5 μg biotinylated goat anti-human IgG (Zymed) in a 100 μL volume of FCM buffer for 45 min, washed, and incubated for 30 min with streptavidin–FITC or streptavidin Cy5/RPE (Dako Cytomation) diluted 1:50 with FCM buffer. Apoptosis and necrosis was measured in the samples either as part of the CD205–IgG flow cytometry assay, or as a separate stain. In the CD205–IgG flow cytometry assay, cells were firstly incubated with 5 μL annexin-V-allophycocyanin (APC) and 5 μL (0.25 μg) 7-amino-actinomycin D (7-AAD; BD Bioscience) in 100 μL CM on ice for 20 min. The cells were washed with CM, and stained with CD205–IgG as detailed previously, using streptavidin–FITC in the detection step. When used as a separate stain, cells were washed in annexin binding buffer (10 mM HEPES pH7.4, 140 mM NaCl, 2.5 mM CaCl_2_), and incubated with 2 μL annexin-V APC and 2 μL 7-AAD for 20 min at room temperature. 300 μL annexin binding buffer was then added to the stain, and the cells analysed by flow cytometry. Annexin-V^−^/7-AAD^−^ cells were taken to be live cells, annexin-V^+^/7-AAD^−^ cells were taken to be primary/early apoptotic cells, and annein-V^+^/7-AAD^+^ cells were taken to be late apoptotic/secondary necrotic cells (when induced to undergo apoptosis with anti-FAS treatment of dexamethasone treatment) and primary necrotic cells (when rapidly induced to undergo unscheduled cell death by heat treatment). Data were acquired using a Becton Dickenson FACSCaliber flow cytometer and CELLQuest software. Data analysis was performed using WinMDI software (Coulter Corporation).

### 2.9 Trypsin sensitivity assay

Live DC2.4 cells were treated with either 1× Versene (Cambrex) or 2× trypsin (Sigma–Aldrich) for 15 min at 37 °C, washed twice with CM, and stained as described. Annexin-V staining demonstrated that trypsin treatment did not induce apoptosis in DC2.4 cells (data not shown).

### 2.10 CD205 endocytosis assay

Anti-CD205 (clone NLDC-145), and anti-ICAM-1 (clone YN1.1), were purified in house from hybridoma supernatants. The isotype controls rat IgG2a and rat IgG2b were purchased from Serotec. YO1 cells were grown on coverslips, and incubated for 30 min at 37 °C with CM with 100 U/mL IFNγ and 30 μg/mL antibody. The cells were washed three times with warm PBS and fixed with 4% paraformaldehyde in PBS for 20 min on ice. The cells were permeablised with 0.1% saponin (Sigma–Aldrich) in PBS for 10 min at room temperature, and then incubated with biotinlylated sheep anti-rat IgG (GE Healthcare) diluted 1:200 with PBS/0.1% saponin on ice for 30 min. The cells were washed with PBS/0.1% saponin three times, and incubated for 20 min with streptavidin–TRITC (Sigma–Aldrich) diluted 1:100 in PBS/0.1% saponin before washing again. The nuclei were stained with 2.5 μg/mL bis-benzimide (Sigma–Aldrich) in PBS for 5 min, washed three times with PBS, and mounted onto microscope slides using fluorescence mounting medium (DAKO). Slides were visualised under a fluorescence microscope (Zeiss Axiovert S100 TV) under the 100× oil immersion objective lens.

### 2.11 Apoptotic cell uptake assays

YO1 cells were seeded into 24-well plates and placed at 37 °C three days prior to use. At confluence, this provided 5 × 10^5^ cells per well. Thymocyte suspensions were labelled with 1 μM CFSE (Sigma–Aldrich) in PBS for 5 min at room temperature, washed three times with CM, and induced to undergo apoptosis for 4 h with 1 μM dexamethasone. Thymocytes were extensively washed with CM before use. 2 × 10^6^ CFSE-labelled dexamethasone treated thymocytes were added to the YO1 cells in the presence of 1.4 μg/mL CD205–IgGs, and incubated for 16 h at 37 °C. Unbound/unengulfed thymocytes were removed by gently washing three times with warmed PBS. The YO1 cells were harvested by incubating for 2 min with 2× trypsin (Sigma–Aldrich), washed with FCM buffer, and analysed by flow cytometry. For BMDC uptake experiments, 1 × 10^6^ CFSE-labelled thymocytes were dexamethasone treated overnight and pre-incubated with 100 μL 18 μg/mL CD205–IgGs for 30 min, and transferred onto 2 × 10^5^ BMDCs in 100 μL CM in 5 mL flow cytometry tubes. The cells were incubated for 4 h at 37 °C, with a control tube incubated at 4 °C to inhibit phagocytosis. The cells were washed with FCM buffer, incubated with Versene (Cambrex) for 20 min, washed again with FCM buffer and analysed by flow cytometry. Unbound/unengulfed thymcoytes were excluded from analysis on the basis of size.

## 3 Results

**Fig. 1:**
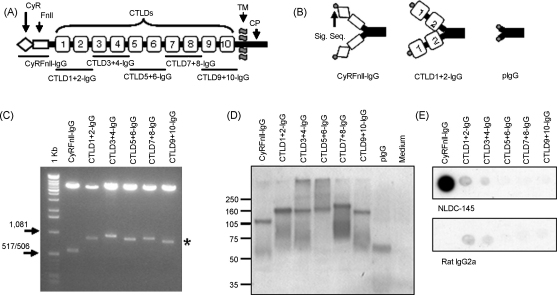
Construction of a panel of CD205-IgG fusion proteins. (A) The structure of the CD205 molecule. Domains are: cysteine-rich domain (CyR), fibronectin type-II domain (FnII), C-type lectin-like domains (CTLDs), transmembrane domain (TM), and cytoplasmic domain (CP). The extracellular domains of the CD205 molecule were cloned as pairs into the signal PIG plus vector, creating a panel of six CD205-IgG fusion proteins. (B) Three types of protein were produced by expression in mammalian cell lines—the CyRFnII-IgG, five pairs of CTLD-IgG fusion proteins, and the signal PIG plus vector IgG1 tail (pIgG) as a control IgG (from expressing the empty vector). The vector-derived CD33 signal sequence (Sig. Seq.) guides the expression of the fusion proteins. (C) Restriction digest of the plasmid constructs using the primer-derived restriction sites. The CD205 insert sequences are highlighted by the asterisk (*). (D) Western blot to detect fusion proteins in transfected CHO cell supernatants developed using anti-human IgG antibody. (E) Dot blot of purified fusion proteins, using NLDC-145 (rat anti-mouse CD205) or an isotype control. The epitope recognised by NLDC-145 is within the CyR domain, and the epitope is conserved in the CyRFnII-IgG fusion protein. Results are representative of two experiments.

We created a panel of six CD205–IgG fusion proteins, depicted in Fig. 1—the CyR and FnII domains paired together (CyRFnII-IgG), and the remaining CTLDs cloned as pairs (CTLD1+2-IgG, CTLD3+4-IgG, CTLD5+6-IgG, CTLD7+8-IgG, and CTLD9+10-IgG). cDNA encoding these domains was amplified from thymic epithelial cell cDNA, by PCR using primers with flanking restriction enzyme sites. The PCR products were inserted into the signal PIG plus vector MCS using these restriction sites; this fused the CD205 domains to a human IgG1 tail and the CD33 signal sequence. The six fusion protein plasmids, plus the empty signal PIG plus vector (pIgG), were used to transfect CHO cells. The fusion proteins were secreted in the culture supernatant, which could either be used directly in flow cytometry experiments, or could be purified via the IgG tail by protein-A affinity chromatography.

**Fig. 2:**
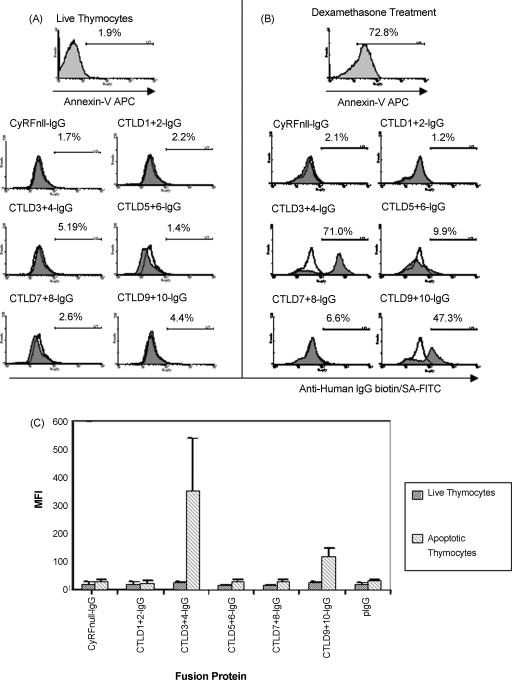
CTLD3+4-IgG and CTLD9+10-IgG bind ligands expressed by dying thymocytes. The fusion protein panel was used to stain either live thymocytes (A), or thymocytes that had been induced to undergo apoptosis by incubation with 1 μM dexamethasone for 16 h (B). Fusion protein binding was fixed with paraformaldehyde, then detected using biotinylated anti-human IgG and streptavidin (SA)–FITC (results representative of 3 experiments). CTLD3+4-IgG and CTLD9+10-IgG strongly bind apoptotic, but not live, thymocytes. The median fluorescent intensities of fusion protein staining from three independent experiments are represented in (C), plotted as mean value + SD. CTLD3+4-IgG staining is consistently stronger than CTLD9+10-IgG staining. Annexin-V histograms—light grey fill is annexin-V staining. CD205-IgG histograms—line is pIgG control staining, dark grey fill is the fusion protein indicated.

We used the CD205–IgG fusion protein panel in a flow cytometry assay to detect the binding of individual panel members to live and apoptotic thymocytes. Thymocytes were used as a model of apoptosis for two reasons; firstly, they are extremely sensitive and undergo apoptotic cell death in response to a wide range of stimuli. Secondly, the thymus itself is a site of high CD205 expression, with the cortical thymic epithelium and medullary DCs expressing CD205 (Jiang et al., 1995). We therefore reasoned that developing thymocytes in contact with the cortical epithelium and medullary DCs express CD205 ligands. We compared the ability of the fusion protein panel to stain untreated, freshly isolated thymocytes, and thymocytes that had been induced to undergo apoptosis by incubation for 16 h with dexamethasone. As seen in Fig. 2, none of the CD205–IgG fusion proteins were able to bind live thymocytes (Fig. 2A). However, CTLD3+4-IgG and CTLD9+10-IgG were able to bind apoptotic thymocytes (Fig. 2B). The interaction of these fusion proteins with their cognate ligand(s) occurs with low affinity, as paraformaldehyde fixation is required to detect the interaction (described in Section 2). No binding was seen with the other CD205–IgG fusion proteins, or with the pIgG control.

**Fig. 3:**
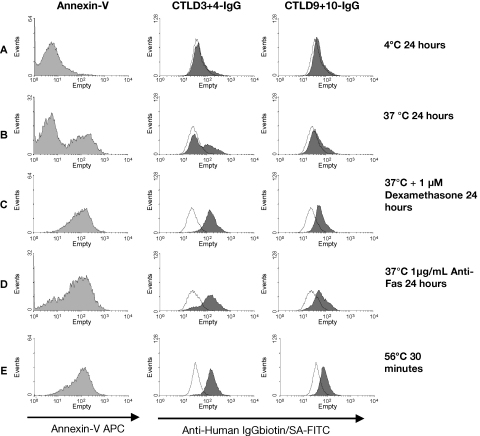
Thymocytes express CD205 ligands in response to multiple pro-apoptotic and pro-necrotic stimuli. Thymocytes were incubated for 24 h at 4 °C to prevent cell death (A), or induced to undergo apoptosis by incubation for 24 h at 37 °C with no extra stimuli (B) or with 1 μM dexamethasone (C), or 1 μg/mL anti-Fas antibody (D). Primary unscheduled necrosis was induced by heat treating freshly isolated thymocytes at 56 °C for 30 min (E). Thymocytes were stained with annexin-V, then washed and stained with CTLD1+2-IgG (control), CTLD3+4-IgG or CTLD9+10-IgG. All of the stimuli tested induced CD205-IgG ligand expression bound by CTLD3+4-IgG and CTLD9+10-IgG. The level of CD205-IgG ligand expression correlates with the level of annexin-V binding. Annexin-V histograms—light grey fill is annexin-V staining. CD205-IgG histograms—line is CTLD1+2-IgG control staining, dark grey fill is the fusion protein indicated (CTLD3+4-IgG or CTLD9+10-IgG). Results are representative of two experiments.

Having established that CTLD3+4-IgG and CTLD9+10-IgG were the only two proteins able to mediate ligand binding, we reduced our reagent panel to these two binding proteins plus a non-binding control region, CTLD1+2-IgG. We then examined whether the expression of CD205 ligand(s) was induced solely by dexamethasone, or whether other pro-apoptotic and pro-necrotic stimuli could induce expression (Fig. 3). Thymocytes were cultured for 24 h at 4 °C to prevent cell death (A), at 37 °C with no additional treatment (B), and with 1 μM dexamethasone (C) or 1 μg/mL anti-Fas antibody (D). All of these stimuli were able to induce both apoptosis and CD205 ligand expression. Furthermore, heating thymocytes at 56 °C for 30 min to induce primary unscheduled necrosis also resulted in the expression of CD205 ligands (E). This demonstrated that the expression of CD205 ligand(s) was a general feature of thymocyte death, rather than a phenomenon specifically induced by steroid treatment. Interestingly, the proportion of cells bound by CTLD3+4-IgG and CTLD9+10-IgG appeared to correlate with the proportion of annexin-V-binding cells.

**Fig. 4:**
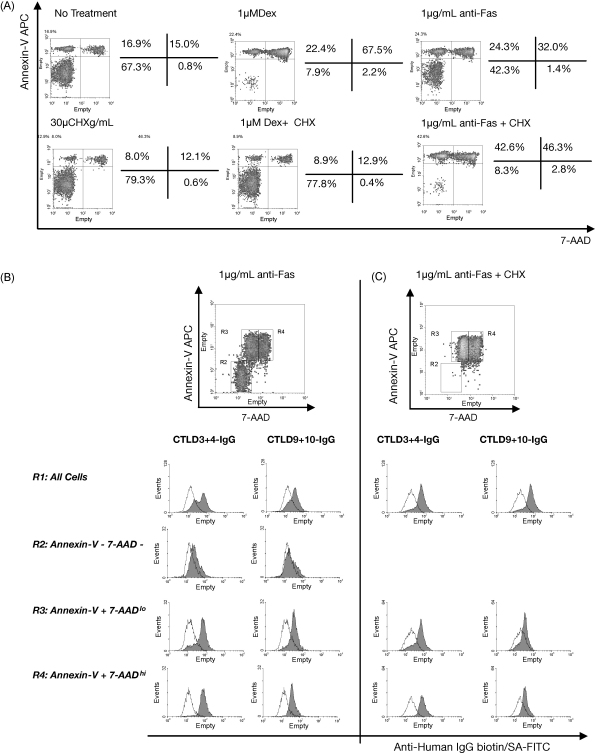
CD205 ligands are expressed by primary apoptotic and secondary necrotic thymocytes, and expression does not require protein synthesis. (A) Thymocytes were cultured at 37 °C with no treatment, dexamethasone (DEX), or anti-Fas antibody, both in the presence and absence of cyclohexamide (CHX), and stained with annexin-V and 7-AAD. Dexmethasone is unable to induce apoptosis in the presence of cyclohexamide, but cyclohexamide treatment enhances apoptosis induced by anti-Fas antibody. (B) Anti-Fas treated thymocytes were stained with 7-AAD and annexin-V, then washed and stained with the CD205-IgGs CTLD1+2-IgG (control), CTLD3+4-IgG or CTLD9+10-IgG. Minimal binding of CTLD3+4-IgG and CTLD9+10-IgG is seen within the annexin-V^−^ 7-AAD^−^ fraction (R2), whereas strong binding of both CTLD3+4-IgG and CTLD9+10-IgG is seen in the annexin-V^+^/7-AAD^hi^ fraction (R4, secondary necrotic cells). Within the annexin-V^+^ 7-AAD^lo^ population (R3, primary apoptotic cells) a large proportion of the cells are bound by CTLD3+4-IgG and CTLD9+10-IgG, whereas a small proportion are not. CD205-IgG histograms—line is CTLD1+2-IgG control staining, dark grey fill is the fusion protein indicated. Results are representative of two experiments.

Next, we examined whether de novo synthesis of proteins is required for CD205-ligand expression during apoptotic cell death. Thymocytes were incubated for 24 h with medium alone, 1 μM dexamethasone, or 1 μg/mL anti-Fas antibody, both in the presence and absence of 30 μg/mL cyclohexamide to inhibit protein synthesis. The thymocytes were stained with annexin-V and 7-amino-actinomycin D (7-AAD) and analysed by flow cytometry to quantify the proportion of cells undergoing apoptosis. As seen in Fig. 4A, cyclohexamide inhibited the ability of dexamethasone to induce apoptosis. This is expected, as dexamethasone is a steroid that requires gene transcription and translation to induce apoptosis. However, cyclohexamide was not able to inhibit the progression of apoptosis induced by ligation of the Fas molecule on thymocytes. We therefore examined the binding of CD205–IgGs to anti-Fas treated cells more closely, using a triple-stain technique.

Fas-treated thymocytes were stained with annexin-V and 7-AAD, and then stained with CTLD3+4-IgG or CTLD9+10-IgG and fixed with paraformaldehyde. The bound CD205–IgGs were detected with FITC-conjugated secondary reagents, and analysed by flow cytometry (Fig. 4B). Live thymocytes (annexin-V^−^ 7-AAD^−^) were weakly bound by CTLD3+4-IgG, and were not bound by CTLD9+10-IgG. Early apoptotic cells (appearing as annexin-V^+^ 7-AAD^lo^ cells in the triple stain where paraformaldehyde fixation is used, and as annexin-V^+^ 7-AAD^−^ cells in the double stain where fixation is not carried out) were bound by both CTLD3+4-IgG and CTLD9+10-IgG (Fig. 4B); both fusion proteins were able to bind more strongly still to the late apoptotic/secondary necrotic population (annexin-V^+^ 7-AAD^hi^). This, together with the data presented in Fig. 3 demonstrates that CD205 is able to bind early apoptotic, late apoptotic/secondary necrotic, and primary necrotic thymocytes; thus CD205 binds ligand(s) expressed during both necrotic and apoptotic cell death. Thymocytes treated with both anti-Fas antibody and cyclohexamide had progressed further through apoptosis than when treated with anti-Fas antibody alone (Fig. 4C)*.* However, the level of CTLD3+4-IgG and CTLD9+10-IgG binding to the primary apoptotic and secondary necrotic thymocyte populations is equivalent to that seen without cyclohexamide treatment. This clearly demonstrated that the expression of CD205 ligand(s) at the surface of apoptotic/necrotic cells did not require *de novo* protein synthesis.

**Fig. 5:**
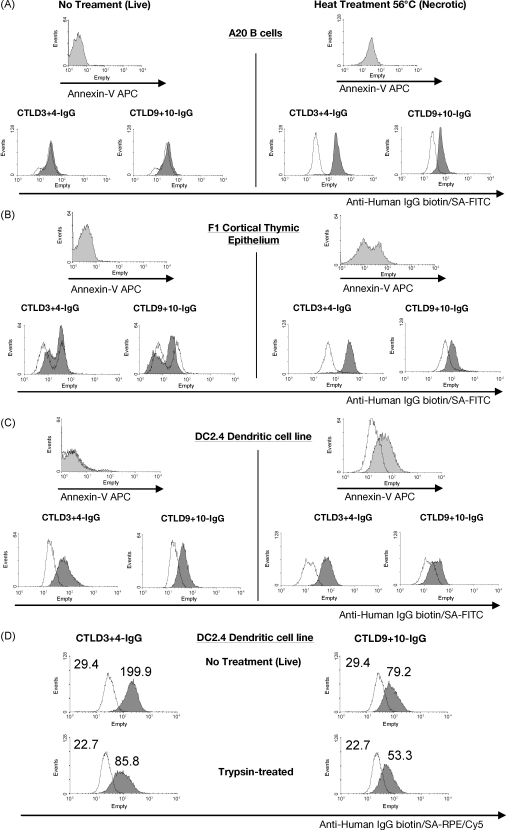
CD205 ligands are expressed by multiple cell types during primary (unscheduled) necrosis, and expressed by live DC2.4 cells. A20 B cells (A) and F1 cortical thymic epithelial cells (B) and DC2.4 cells (C) were heat treated for 30 min at 56 °C to cause rapid primary (unscheduled) necrosis, and stained with annexin-V and the CD205-IgGs CTLD1+2-IgG (control), CTLD3+4-IgG or CTLD9+10-IgG. A20 and F1 cells are bound by CTLD3+4-IgG and CTLD9+10-IgG in a primary necrotic state. DC2.4 cells are bound by CTLD3+4-IgG and CTLD9+10-IgG both when the cells are live, and when induced to undergo primary unscheduled necrosis. Results are representative of two experiments. (D) DC2.4 cells were treated for 10 min with Versene or with trypsin before staining with the control CTLD1+2-IgG, CTLD3+4-IgG or CTLD9+10-IgG. The fluorescent intensity of staining with CTLD3+4-IgG and CTLD9+10-IgG is reduced by pre-incubation with trypsin. Annexin-V histograms—light grey fill is annexin-V staining. CD205-IgG histograms—line is CTLD1+2-IgG control staining, dark grey fill is the fusion protein indicated.

We next looked at whether CTLD3+4-IgG and CTLD9+10-IgG could bind dying cells from other cell lineages. We induced primary necrosis in A20 B cells and F1 thymic epithelial cells by heating to 56 °C for 30 min. Both of these cell lines were specifically bound in their necrotic state by CTLD3+4-IgG and CTLD9+10-IgG (Fig. 5A and B). This demonstrated that the expression of CD205 ligand(s) during cell death was a multilineage phenomenon, and one that occurs both within the thymus and the periphery. However when we stained the immortalised DC cell line DC2.4 we found that both live *and* necrotic cells were bound by CTLD3+4-IgG and CTLD9+10-IgG (Fig. 5C). This was surprising, as no other cells tested had expressed CD205 ligand(s) when in a live, non-apoptotic/non-necrotic state. Staining of the JAWSII immortalised APC line demonstrated that this too expresses CD205 ligand(s) when live and non-apoptotic (data not shown), indicating that the expression of CD205 ligand(s) on live cells may be a feature of professional phagocytes. We next treated DC2.4 cells with trypsin prior to staining with CD205–IgGs, to test whether trypsin-sensitive proteins are involved in the recognition of ligands by CD205. As seen in Fig. 5D, CTLD3+4-IgG and CTLD9+10-IgG bind a trypsin-sensitive ligand(s) on DC2.4 cells.

**Fig. 6:**
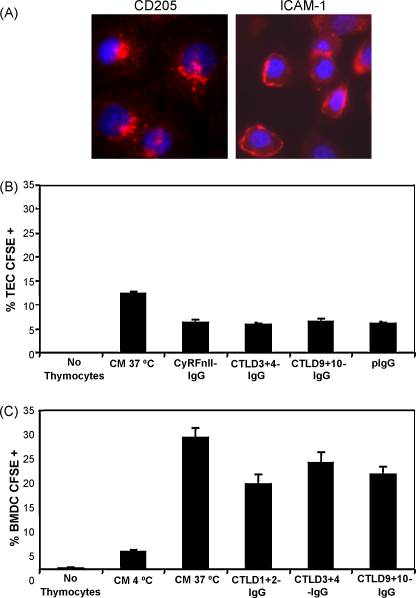
CD205-IgGs are unable to block the uptake of apoptotic thymocytes by YO1 cortical thymic epithelial cells (TEC) or by bone marrow derived dendritic cells (BMDCs). (A) The YO1 cell line was incubated with anti-CD205 and anti-ICAM-1 antibodies, which were allowed to internalise at 37 °C. The cells were fixed, permeablised, stained with TRITC-labelled secondary reagents and bis-benzimide nuclear stain, and analysed by fluorescence microscopy. The non-endocytic molecule ICAM-1 remains at the cell surface, whereas the endocytic CD205 molecule localises to the perinuclear region of the cell (results representative of 2 experiments). (B) YO1 cells were incubated with dexamethasone treated CFSE-labelled thymocytes for 16 h at 37 °C with 1.4 μg/mL CD205-IgGs, and analysed by flow cytometry. The CTLD3+4-IgG and CTLD9+10-IgG fusion proteins were not able to block uptake above background levels seen with CyRFnII-IgG and pIgG controls. CM, complete medium. Results are mean values of triplicate samples + SD. (C) CD205-IgGs (18 μg/mL) were similarly unable to block the uptake of CFSE-labelled apoptotic cells by BMDCs. Results are mean values of triplicate samples + SD. Results are representative of two experiments.

Having demonstrated that CTLD3+4-IgG and CTLD9+10-IgG are capable of binding dying thymocytes (and indeed other cell types), we investigated whether these CD205–IgGs could block the uptake of apoptotic cells by the CD205-expressing cortical thymic epithelial cell line, YO1 (Williams et al., 1996). The cell line is conditionally immortalised at 33 °C by a temperature-sensitive SV40 T antigen gene under the control of the MHC H-2Kb promoter, and can be incubated at 37 °C prior to experiments to provide a more physiologically representative cortical thymic epithelial cell. Although CD205 is endocytic on immature DCs, we have recently demonstrated that this is not the case on mature DCs (Butler et al., 2007). We therefore tested whether CD205 is endocytic on cortical thymic epithelial cells. YO1 cells were moved to 37 °C for three days, and then incubated with anti-CD205 antibody or anti-ICAM-1 antibody at 37 °C. The cells were washed, fixed, and permeablised, and antibody staining was detected using TRITC-conjugated secondary reagents. As seen in Fig. 6A, the non-endocytic cell surface molecule ICAM-1 remains at the surface of the YO1 cell, whereas CD205 is endocytosed and localises to the perinuclear region of the cell. This demonstrates that CD205 is indeed endocytic on cortical thymic epithelium.

Next, we incubated dexamethasone-treated CFSE-labelled thymocytes with YO1 cells in the presence of CD205–IgGs for 16 h at 37 °C, before washing the free thymocytes from the YO1 cells and analysing by flow cytometry. As seen in Fig. 6B, CTLD3+4-IgG and CTLD9+10-IgG were unable to block the uptake of apoptotic thymocytes by YO1 cells. However, the YO1 line itself did not show a high level of phagocytic activity. We therefore tested the ability of CD205–IgGs to block phagocytosis by BMDCs, which express CD205 and are known to phagocytose apoptotic cells and present apoptotic cell-derived antigens on MHC class II (Inaba et al., 1995). As seen in Fig. 6C, our CD205–IgG constructs were unable to block the uptake of apoptotic thymocytes by BMDCs, or the immortalised JAWS II APC line (data not shown).

## 4 Discussion

In this study, we have used a panel of CD205–IgG fusion proteins to determine the distribution of CD205 ligands on murine cells. We show that CTLDs 3+ 4 and 9+ 10 are able to bind cells in two different situations; whereas the cloned DC/APC cell lines DC2.4 and JAWSII (data not shown) express CD205 ligand(s) when live, thymocytes, B cells and cortical thymic epithelial cells only expressed CD205 ligand(s) when undergoing apoptosis and necrosis. Our data therefore suggest that CD205 acts as a recognition receptor for dying cells from multiple cell lineages, and hence may participate in the recognition of apoptotic cells during the uptake and subsequent presentation of apoptotic cell-derived self-antigens, thus playing a key role in the development of immunological tolerance.

In phagocytic cell systems, the recognition and engulfment of apoptotic cells is known to involve the engagement of a broad spectrum of recognition receptors at the surface of the phagocyte recognising changes at the surface of the apoptotic cell (Lauber et al., 2004). Early in apoptosis, phoshpatidylserine (PS) is externalised from the interior of the phospholipid bilayer, and is recognised by the receptor/bridging molecule pairs PS Receptor/annexin I (Arur et al., 2003), β2-glycoprotein-I receptor/β2-glycoprotein-I (Balasubramanian et al., 1997), Mer/growth arrest specific-6 (Gas6) (Ishimoto et al., 2000; Scott et al., 2001), and αvβ3/5 integrins/milk-fat globule-EGF-factor 8 (MFG-E8) (Hanayama et al., 2002). Oxidised lipids are recognised by scavenger receptors (SRs) such as SR-A (Platt et al., 1996), lectin-like oxLDL-receptor I (LOX1) (Oka et al., 1998), and CD36 (Ren et al., 1995) (which also forms a bridged recognition system with thrombospoindin-1 and αvβ3/5 integrins (Savill et al., 1992; Rubartelli et al., 1997)). Altered sugar residues on apoptotic cells are bound by mannose binding lectin (MBL), surfactant proteins A/D, and C1q. These are in turn bound by the CD91/calreticulin (CRT) complex on phagocytes (Vandivier et al., 2002). CRT is also translocated to the surface of apoptotic cells, and is bound by LDL-receptor-related protein (LRP) on the phagocyte (Gardai et al., 2005). Our data indicate that CD205 may contribute to this diverse, redundant system of apoptotic cell recognition.

It was initially surprising to find that two different CTLD domain-pairs have ligand binding activity, as the other members of the MR-family contain only one CTLD domain that is primarily responsible for ligand binding, although flanking CTLDs can provide increased binding avidity (East and Isacke, 2002). However CD205 is unusual within the family as it contains 10 rather than 8 CTLDs, and our data suggest that these extra domains (CTLD9+10 are a duplication of CTLD7+8 (Taylor, 1997)) do play a functional role in ligand binding. It is unlikely that both of the CTLDs within each domain pair mediate ligand binding; however due to the nature of our CD205–IgG domain-pair library the exact domain responsible for binding in each case has not been determined. Since CTLD3+4-IgG staining is consistently higher than CTLD9+10-IgG staining, this suggests that CTLD3+4-IgG and CTLD9+10-IgG may either bind distinct ligands or the same ligand with different affinities.

The finding that the proportion of cells expressing CD205 ligands was higher in secondary apoptotic/necrotic cells (annexin-V^+^ 7-AAD^hi^) than in primary apoptotic populations (annexin-V^+^ 7-AAD^lo^) suggests that progression of cells through death pathways results in the accumulation of CD205 ligand(s) at the surface of the dying cell. Furthermore, ligand expression during cell death does not require *de novo* protein synthesis. Taken together, these data suggest that CD205 ligand(s) may be translocated from the interior to the exterior of the cell during apoptosis. This is indeed the case for other molecules upregulated during apoptosis such as PS. Additionally, protein molecules normally resident within the endoplasmic reticulum (ER), such as calreticulin, calnexin, KDEL receptor, and the cellular heat shock proteins HSP-70 and HSP-90 are translocated to the surface of apoptotic cells (Franz et al., 2007). Calreticulin is additionally expressed at the surface of phagocytes (Gardai et al., 2005), a situation that mirrors our finding that CD205 ligand(s) are expressed by live DC2.4 cells.

Using live DC2.4 cells, we were able to demonstrate that the ligand(s) bound by CD205 are trypsin-sensitive and thus involves proteins. This could either be by the direct recognition of a protein on the apoptotic cell, or the indirect recognition via a protein bridging molecule. However, we have not been able to identify the ligands bound by CTLD3+4-IgG and CTLD9+10-IgG, as attempts to purify the ligands from either DCs or apoptotic/necrotic cells were unsuccessful (data not shown). This is likely to be due to the low affinity of the domain-pairs for their ligands, illustrated by the fact that staining could only be revealed following fixation with paraformaldehyde. This may be an inherent feature of the native CD205 molecule, or may be because the whole backbone of 10 CTLDs is required for maximal binding avidity. Indeed, the related MR is able to bind ligands through the CTLD4 region, but CTLDs4–8 are required for maximal binding to yeast mannan (Taylor and Drickamer, 1993). Furthermore, our CTLD3+4-IgG and CTLD9+10-IgG fusion proteins were unable to block the uptake of apoptotic cells by BMDCs or YO1 cortical thymic epithelial cells. Although this may be due to functional redundancy between phagocytic receptors (CD205, CD36 and β5-integrin knockout mice *all* show no defects in apoptotic cell uptake and antigen presentation (Iyoda et al., 2002; Schulz et al., 2002)), it is more likely that our fusion protein dimers do not have sufficient affinity for their ligands to compete with multivalent CD205 on the phagocyte surface.

Out data differ from those of Small and Kraal (2003), who showed blocking of apoptotic thymocyte uptake by the antibody NLDC-145. In our hands, NLDC-145 did not block the uptake of apoptotic thymocytes by either YO1 cells or BMDCs (data not shown). Moreover, NLDC-145 binds an epitope within the CyRFnII region of the molecule, and our data with CD205–IgG fusion proteins have shown that this region of CD205 does not play a role in binding of apoptotic cells. It is however possible that our BMDCs and the YO1 cell line rely on different receptors for cell uptake compared to the BA/10 cell line used by Small and Kraal.

The exact role of CD205 in apoptotic cell recognition and uptake remains to be fully elucidated. It is unlikely that CD205 itself engages the phagocytic machinery required for cell engulfment; indeed CD205 is expressed by non-phagocytic cell types such as T cells (Kato et al., 2006). It is more likely that CD205 forms part of a recognition synapse with other receptors capable of engaging the phagocytic machinery, such as PS-receptor (Somersan and Bhardwaj, 2001). CD205 may play a role in guiding the intracellular trafficking of phagosomes to late endosomes, due to the EDE targeting motif in its intracellular tail (Mahnke et al., 2000). The ability of CD205 to bind apoptotic cells may act to include the molecule within the phagosome, rather than dictating the formation of the phagosome itself. CD205 may additionally mediate the uptake of smaller vesicular structures such as apoptotic blebs; characterisation of the ligands bound by CD205 would allow a more comprehensive view of its physiological roles.

The uptake of dying cells by CD11c+ CD8+ CD205+ DCs and the subsequent presentation and cross-presentation of antigens to CD4+ and CD8+ T cells is of crucial importance in both the induction of immunity to intracellular viral pathogens, and the induction and maintenance of tolerance to self-antigens (Belz et al., 2002c, 2004; Heath et al., 2004). Our study has demonstrated that CD205 acts as a recognition receptor for dying cells. As such, CD205 is likely to be an important molecule in the induction and tolerance to self both intrathymically and within the periphery. Within the thymus, CD205 may aid the recognition and processing of dying cells by cortical thymic epithelium, and therefore influence the acquisition and presentation of antigens for positive selection (Anderson et al., 1999). It may additionally contribute to the phenomenon of “antigen spreading” in the thymus, whereby medullary dendritic cells are able to acquire peripheral antigens expressed by medullary epithelial cells, possibly by the engulfment of apoptotic bodies or exosomes (Gallegos and Bevan, 2004; Gray et al., 2007). This leads to efficient deletion of autoreactive T cell clones by DCs. Within the periphery, CD205-mediated acquisition of apoptotic/necrotic material has the potential to influence both peripheral tolerance and immunity.

## Figures and Tables

**Table 1:** PCR Primers used for cloning the CD205-IgG fusion protein panel. PCR primers were designed with flanking restriction enzyme sites (bold typeface). Digestion with the indicated restriction enzymes allowed direction insertion into the signal PIG plus vector's multiple cloning site.

CD205 domains	Size (bp)	Primer sequence (5′–3′)	Restriction enzyme
CyRFnII	555	Forward: GCCCTA***AAGCTT***TCTGAGAGCTCAGGTAAT	Hind III
		Reverse: ATGACCT***GGATCC***ATTGGTAGTAG GC GAT	Bam HI
CTLD1+2	855	Forward: ATATCG***CTCGAG***TGTGAAGGTAACTGGGAA	Xho I
		Reverse: CGAATC***TCTAGA***CAGCTTATCCGACTCTGC	Xba I
CTLD3+4	930	Forward: ATTAATAAA***GGTACC***TGTCCGCCAGACGAG	Kpn I
		Reverse: TACT***GGATCC***TCTCCATGGCGCTCTGGATT	Bam HI
CTLD5+6	861	Forward: ATATGG***AAGCTT***ATTCATGGGCCCCCAGTT	Hind III
		Reverse: GCGGCGC***TCTAGA***TTCAGTCT CTGAGTATT	Xba I
CTLD7+8	891	Forward: AATTTAT***AAGCTT***GACGGACAGCCCTGGGA	Hind III
		Reverse: CACCTG***TCTAGA***GCCATTGTGTTTGTCCTC	Xba I
CTLD9+10	819	Forward: ATGCAG***AAGCTT***ACCCTGCCACAGTTCATT	Hind III
		Reverse: AATT***GGATCC***ATGTCCGGGCTCAGAGGAAT	Bam HI

## Footnotes

1 Abbreviations used: MR, mannose receptor; PLA2R, phospholipase A2 receptor; CyR, cysteine-rich domain; FnII, fibronectin type II domain; CTLD, C-type lectin like domain; CM, culture medium; PS, phosphatidylserine; 7-AAD, 7-amino-actinomycin D; APC, allophycocyanin.
